# Fenugreek seed and cape gooseberry leaf extracts as green corrosion inhibitors for steel in the phosphoric acid industry

**DOI:** 10.1038/s41598-022-26757-z

**Published:** 2022-12-23

**Authors:** A. M. Abdel-Gaber, A. Ezzat, M. E. Mohamed

**Affiliations:** Chemistry Department, Faculty of Science, Alexandria University, Alexandria, Egypt

**Keywords:** Chemistry, Engineering, Materials science

## Abstract

Phosphoric acid is the core material for the fertilizer industry; however, it is incredibly corrosive to manufacturing plants’ structures, mainly steel. Corrosion is one of the most severe problems encountered during phosphate fertilizer manufacturing. Recently, plant extracts have been commonly used as corrosion inhibitors because they are cheap and environmentally friendly. Steel corrosion in a 20% aqueous phosphoric acid solution in the absence and presence of fenugreek seed (Fen) or cape gooseberry leaf (CgL) extracts was investigated using the electrochemical impedance spectroscopy technique, potentiodynamic polarization measurement, scanning electron microscope, and quantum chemical calculations. Fourier Transform Infrared, FTIR, was used to identify the functional groups in Fen and CgL extracts. The inhibition efficiency for steel in 20% aqueous phosphoric acid was roughly equal to 80% for 0.4 g/L CgL and 1.2 g/L Fen extracts. A scanning electron microscope showed that the chemical constituents of extracts block the surface roughness of steel, decreasing the corrosion rate. The activation parameters indicated the effectiveness of the extracts at a higher temperature. Measurements of the potential of zero charges showed that the steel surface is positively charged in the phosphoric acid solution. Quantum chemical computations were also employed to examine the corrosion inhibition mechanisms of the natural extracts.

## Introduction

Mild steel is significant in many industries since it is used in various equipment. Its widespread use may be due to favorable qualities such as strength, hardness, and low cost. Mild steel can be exposed to corrosive environmental conditions, making it susceptible to corrosion and thereby deteriorating its properties.

Phosphate rock (PR) and phosphoric acid (PA) are the core materials for the fertilizer industry; however, they are incredibly corrosive to manufacturing plants’ structures, mainly steel. Corrosion products and rust can contaminate fertilizers and cause severe mechanical damage and degradation to plant machinery and facilities^1^. Phosphoric acid is highly corrosive to steel. The 20% phosphoric acid is one of the concentrations utilized in manufacturing fertilizers. Steel materials used in H_3_PO_4_ solutions must be adequately protected. Corrosion inhibitors play a critical role in fertilizer production, storage, and handling, ensuring the long-term viability of equipment and industrial structures. Organic compounds and plant extracts are the most commonly used steel inhibitors in HCl and H_2_SO_4_^2–10^.

The inhibitory effects of Zanthoxylum alatum plant extract on mild steel corrosion in 20, 50, and 88% aqueous orthophosphoric acid were evaluated utilizing weight loss and electrochemical impedance spectroscopy. In 88% phosphoric acid, Zanthoxylum alatum extract suppresses steel corrosion more efficiently than 20% phosphoric acid^11^. 800 ppm of an alcoholic extract of Pisidium guajava (guava) leaves reduced steel corrosion by 89% in a 1.0 M phosphoric acid solution^12^. The Apricot juice has a maximum inhibition efficiency of 75%^13^. Guar gum was discovered to be a promising carbon steel inhibitor in 2.0 M H_3_PO_4_. Maximum inhibition values of 95.9% were obtained at 1.0 g/L Guar gum concentration^14^. Eucalyptus leaf extract was shown to be a more effective corrosion inhibitor in 0.5 M H_2_SO_4_ solutions than in 0.5 M H_3_PO_4_ solutions^15^. The effect of fenugreek leaf and aqueous seed extracts on the corrosion of mild steel in HCl and H_2_SO_4_ solutions has been investigated. Both fenugreek leaf and seed extracts had higher inhibitory activity in the HCl solution than in the H_2_SO_4_ solution^16^. The inhibitory action of lemon peel and fenugreek leaf extracts on mild steel corrosion in 1 M HCl has been examined. The results reveal that both extracts have good inhibition characteristics^17^. The corrosion inhibition efficiency of acid extract of dried Indian gooseberry leaves for mild steel in 1 N HCl medium was tested. The results demonstrated that gooseberry leave is a good mixed-type corrosion inhibitor with an efficiency of 87.9% at 2% v/v inhibitor concentration^18^. The inhibition efficiency of Chinese gooseberry fruit shell extract as a green and cheap corrosion inhibitor for mild steel in an acidic solution was evaluated by weight loss experiments at different temperatures, and the results disclosed that during a 5-h immersion at 25 °C, the efficiency increased to 94% for a 1000 ppm sample^19^.

The use of toxic chemicals as corrosion inhibitors for steel in phosphoric acid is limited due to environmental issues; many countries launched major green chemistry innovations in response to the Paris climate change agreements. Few studies were performed to investigate using natural product extracts as corrosion inhibitors for steel in phosphoric acid^3–7^. Furthermore, the use of Fenugreek seed (Fen) and Cape gooseberry leaf (CgL) extracts as corrosion inhibitors for steel in phosphoric acid solutions has not been reported. As a result, the inhibitory effects of the Fen and CgL on steel corrosion were investigated in a solution simulating that used in the fertilizer industry (20% phosphoric acid at 30 °C). Various techniques such as electrochemical impedance spectroscopy, potentiodynamic polarization measurements, Fourier Transform Infrared, scanning electron microscopy, and density functional theory were used. Thermodynamics and adsorption considerations were also studied.

## Experimental studies

### Solution preparation

Dilutions of analytical reagent-grade 85% phosphoric acid, Scharlau chemical industry, were used to make 20% phosphoric acid solutions. Fenugreek seed (Fen) and Cape gooseberry leaf (CgL) solution were extracted, as reported by Abdel-Gaber et al.^20^. The solubility of Fen and CgL in 20% phosphoric acid solution is 8.4 g/L and 6.3 g/L at 30 °C respectively. Before each experiment, an appropriate volume of extract solution and a definite volume of 85% phosphoric acid is added to double distilled water in a 100 mL measuring flask and diluted to obtain a solution of 20% phosphoric acid and the required concentration of extract. Plant materials were collected in spring (March) in accordance with institutional, national, and international guidelines and legislation.

### Electrochemical studies

The frequency response analyzer (FRA)/potentiostat, ACM instrument UK) was utilized to measure electrochemical impedance spectroscopy (EIS) and potentiodynamic polarization curves. The cell setup and experimental conditions were similar to those previously mentioned^21^. The working electrode was made of mild steel with the following chemical composition (wt%) (P: 0.005, S: 0.001, Si: 0.260, Mn: 0.710, C: 0.164, and Fe: 98.86). A steel plate of cylindrical shape was encapsulated in Teflon, with only one surface exposed to the test solution. The exposed area (0.36 cm^2^) was mechanically abraded with a series of emery sheets of varying degrees, beginning with coarse (120 grade) and progressing to fine (800 grade). The data were obtained in a three-electrode mode electrochemical cell using a platinum rod (0.36 cm^2^) and silver-silver chloride as counter and reference electrodes. The frequency range for EIS measurements was 0.1 to 3 × 10^4^ Hz with an applied potential signal amplitude of 10 mV around the rest potential. Polarization curve measurements were obtained at a scan rate of 30 mV/min using a potential range of ± 300 mV around the equilibrium potential. Experiments were triple checked to ensure that the measurements were accurate. The standard deviation, SD, of the inhibition efficiency of the 3 measurements was determined and reported.

### Fourier transform infrared (FTIR) analysis

FTIR analysis of the *Fen* and *CgL* extracts was achieved by FTIR 8400S Shimadzu in the spectral region between 4000 and 400 cm^–1^.

### Scanning electron microscopy (SEM)

In the absence and presence of *Fen* and *CgL* extract, abraded steel samples were immersed in a 20%t H_3_PO_4_ acid solution for 1 h. After that, the steel samples were dried at room temperature. The SEM (SEM; model: JSM-200 IT, JEOL) was used to capture micrographs of the steel surfaces.

### Theoretical studies

Optimized structure of the major active ingredients of *Fen* and *CgL* extracts obtained at the B3LYP methods using quantum calculation-based density function theory (DFT). GaussView 6.0.16 was used to calculate the optimized structure's quantum chemical parameters using DFT with the B3LYP/6-311G(+) basis set, as this basis set is recognized as one of the basis sets that provides more precise results in resolving the geometries and electronic properties for many organic compounds. All the DFT calculations are performed under an aqueous phase.

## Results and discussion

### FTIR measurements

**Figure 1:**
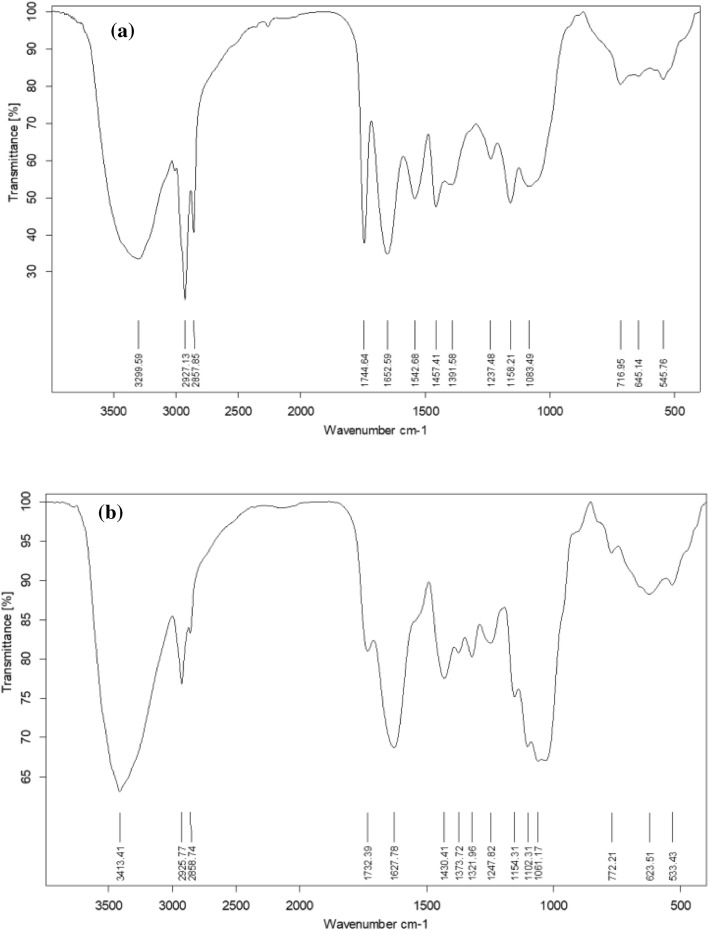
FTIR spectra of (**a**) *Fen* and (**b**) *CgL* extracts.

The FTIR measurements are utilized to characterize the functional groups in *Fen* and *CgL* extracts. Table 1 and Fig. 1 show the band assignments and FTIR spectrum bands for *Fen* and *CgL* extracts. Figure 1a depicts the FTIR spectra of *Fen* seed extract, which show an absorption peak at 3299 cm^−1^ due to the indole ring’s N–H stretching vibrations, absorption peaks at 2927 and 1744 cm^−1^ because of the aliphatic C–H bond, and the carboxylic acid group's C=O group, respectively^22^. These bands correspond to l-tryptophan bands, one of the chemicals extracted from *Fen* seed^23–25^.

**Table 1:** FTIR band assignments for *Fen* and *CgL* extracts.

*Fen* extract		*CgL* extract	
Band, cm^−1^	Assignment	Band, cm^−1^	Assignment
3299	NH stretching	3413	OH of phenolic compound
2927	C–H absorption bands	2925	The aromatic ring C–H stretching
2857	CH_2_ symmetric stretching vibration	2858	C–H symmetric vibration
1744	C=O stretching (e.g., in –COOH groups of amino acids)	1732	*C*=*O* stretching vibration of a carboxyl group
1652	=C–H stretching vibrations	1627	C=C vibration
1542	Aromatic –C=C stretching vibrations	1430	Aromatic C=C bonds
1457	C–H bending (scissoring)	1373	C–O–H bending
1391	COO^−^ symmetric stretch	1321	C–H deformation
1237	C–O stretching band	1247	Phenolic C−O stretching vibrations
1158	C–C/C–N stretching	1154	The C–O–C bridge unsymmetric stretching
1083	In-plane deformation of C–H bonds	1102	C–O–C Cyclic ether
716	Aromatic C–H bending	1061	C–O stretching

**Figure 2:**
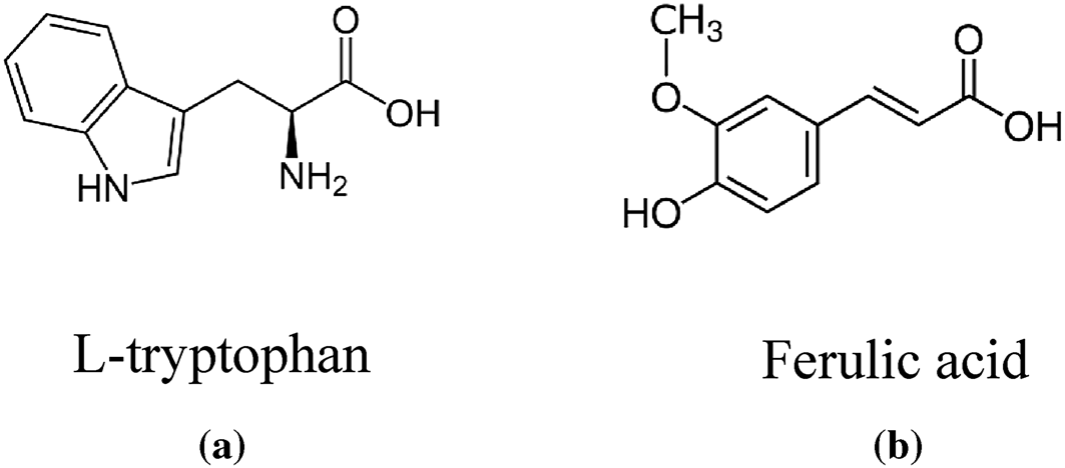
Major chemical constituents of (**a**) *Fen* and (**b**) *CgL* extracts.

The FTIR spectrum of the CgL leaf extract, Fig. 1b, shows the absorption bands for OH of the phenolic compound at 3413 cm^−1^^26^. At 1732 cm^−1^, the stretching vibration of C=O of the carboxyl group occurs, the aromatic ring's C–H stretching vibration at 2925 cm^−1^, C=C vibration at 1627 cm^−1^, aromatic C=C bond at 1430 cm^−1^, unsymmetric stretching of the C–O–C bridge bond and C–O–C cyclic ether at 1154 and 1102 cm^−1^, respectively^27^. These bands are similar to those of ferulic acid, isolated from CgL leaf extract^28,29^. Figure 2 shows the major chemical constituents of Fen and CgL extracts.

### Potentiodynamic polarization measurements

**Figure 3:**
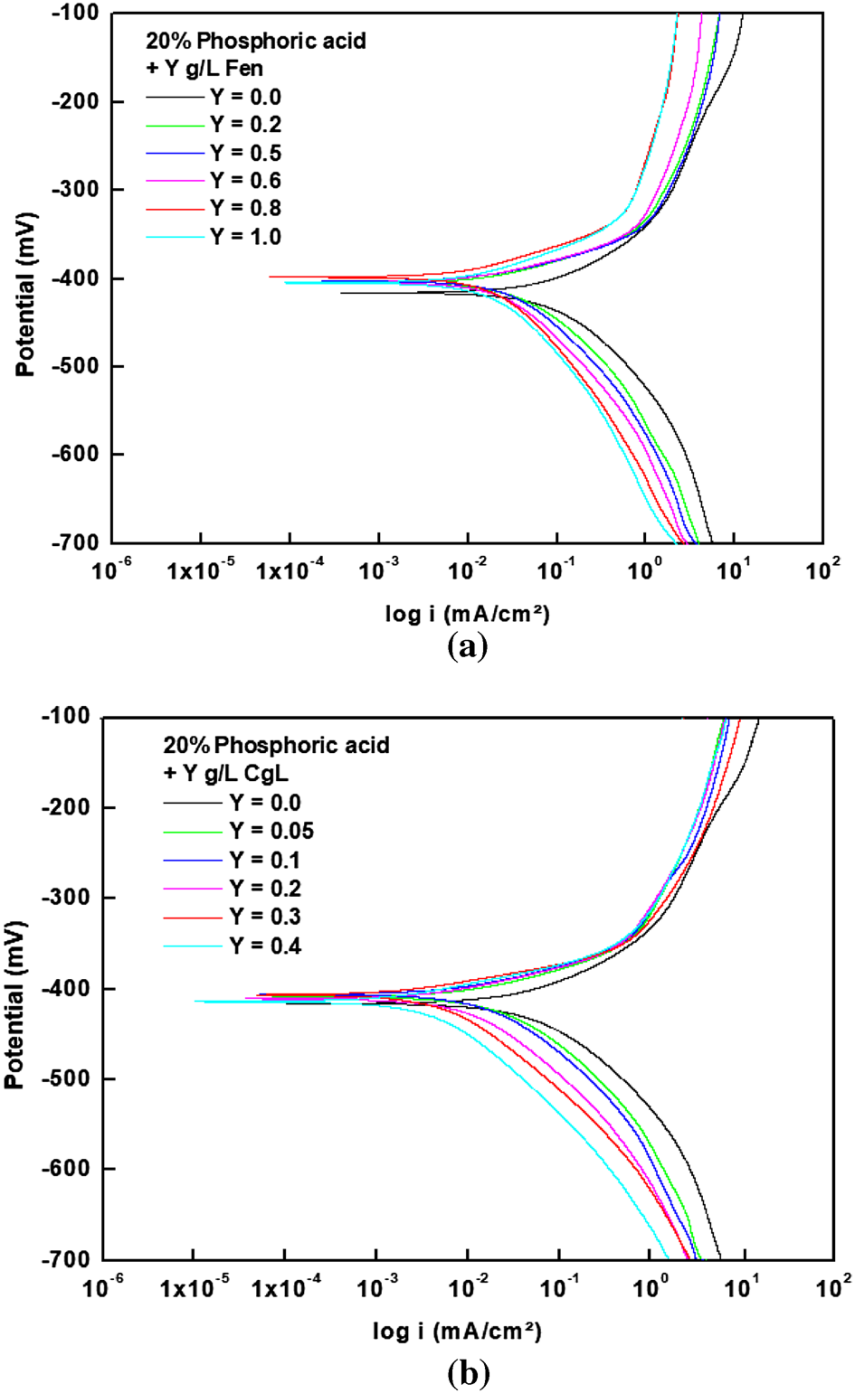
Potentiodynamic polarization plots of steel in 20% phosphoric acid in the absence and presence of (**a**) *Fen* extract and (**b**) *CgL* extract.

Figure 3a indicates that *Fen* extract influences the anodic and cathodic parts of polarization curves, suggesting that it behaves as a mixed-type inhibitor for steel in the phosphoric acid solution. According to Fig. 3b, *CgL* extract acts as a cathodic-type inhibitor, retarding only the hydrogen evolution process. It is obvious that both the anodic and cathodic polarization curves are linear, which means that the corrosion process is under activation control^3^. In air-saturated solutions with pH less than about 3.0, the hydrogen evolution reaction is generally the major cathodic reaction of steel^15^.

1 $${\text{2H}}^{ + } + {\text{ 2e}}^{ - } \to {\text{H}}_{{2}}$$

Self-catalytic processes have been proposed to explain the anodic dissolution of steel in acidic solutions^16^.

2 $${\text{Fe }} + {\text{ OH}}^{ - } \to {\text{Fe}}\left( {{\text{OH}}} \right)_{{{\text{ads}} }} + {\text{ e}}^{ - }$$

3 $${\text{Fe }} + {\text{ Fe}}\left( {{\text{OH}}} \right)_{{{\text{ads}}}} + {\text{ OH}}^{ - } \to {\text{Fe}}\left( {{\text{OH}}} \right)_{{{\text{ads}}}} + {\text{ FeOH}}^{ + } + {\text{2e}}^{ - }$$

4 $${\text{FeOH}}^{ + } + {\text{ H}}^{ + } \to {\text{Fe}}^{{{2} + }} + {\text{ H}}_{{2}} {\text{O}}$$

In water, H_3_PO_4_ is known to be partly dissociated into H^+^ and a series of anion ions (H_2_PO_4_^−^, HPO_4_^2^, PO_4_^3−^). As a result, iron dissolution in phosphoric acid is followed by forming a stable black iron phosphate film on the metal surface^7^.

5 $${\text{3Fe}}^{{{2} + }} + {\text{ 2PO}}_{{4}}{^{{{2} - }}} \to {\text{Fe}}_{{3}} \left( {{\text{PO}}_{{4}} } \right)_{{2}}$$

Table 2 shows the electrochemical polarization parameters. The electrochemical polarization kinetics parameters were obtained by applying the Tafel extrapolation method to both anodic and cathodic polarization lines. The polarization resistances were calculated from the corrosion current density, i_corr_, and the anodic and cathodic Tafel slopes (β_a_ and β_c_) using Eq. (6).

6 $${\text{R}}_{{\text{p}}} = \left( {{1}/\left( {{2}.{3} \times {\text{ i}}_{{{\text{corr}}}} } \right)} \right)\left( {\upbeta_{{\text{a}}} \upbeta_{{\text{c}}}/\upbeta_{{\text{a}}} + \upbeta_{{\text{c}}} } \right)$$

**Table 2:** Electrochemical polarization parameters of steel in 20% phosphoric acid in the absence and presence of *Fen* and *CgL* extracts.

	Conc., g L^−1^	E_corr_, mV	β_a_, mV/decade	− β_c_, mV/decade	i_corr_, mA/cm^2^	R_p_, Ω cm^2^	η	SD
*Fen* extract	0.00	− 493	307	149	6.21	7.01	–	–
	0.20	− 462	218	191	2.95	14.98	52.45	1.01
	0.40	− 475	209	157	2.37	16.42	61.81	0.71
	0.50	− 482	202	131	2.18	15.82	64.81	0.68
	0.60	− 487	208	124	1.68	20.08	72.86	0.57
	0.70	− 487	198	120	1.57	20.66	74.74	0.48
	0.80	− 498	278	138	1.43	28.00	76.88	0.78
	0.90	− 498	244	147	1.38	28.86	77.71	0.52
	1.00	− 498	262	144	1.35	29.89	78.20	0.65
	1.20	− 498	241	157	1.34	30.80	78.47	0.72
*CgL* extract	0.00	− 493	307	149	6.20	7.01	–	–
	0.05	− 468	188	107	2.29	12.93	63.02	0.98
	0.10	− 473	181	105	2.05	14.07	66.93	0.67
	0.20	− 483	181	112	1.57	19.13	74.72	0.59
	0.30	− 500	177	95	1.48	18.14	76.20	0.67
	0.35	− 507	182	104	1.36	21.13	78.03	0.85
	0.40	− 517	190	110	1.24	24.39	80.01	0.24

The inhibition efficiency, η, of the extracts was calculated from i_corr_ utilizing Eq. (7).

7 $$\upeta = \left( {\left( {{\text{i}}_{{\text{o}}} - {\text{i}}_{{{\text{corr}}}} } \right){\text{/i}}_{{\text{o}}} } \right) \times 100$$

In this equation, i_o_ and i_corr_ are the corrosion current densities in the absence and presence of the extract, respectively.

The tabulated data shows that the corrosion potential values, E_corr_, vary slightly as the concentrations of both extracts increase. As a result, these extracts have the potential to be employed as pickling inhibitors. Furthermore, in the presence of the extracts, the corrosion current density (i_corr_) decreases while the polarization resistance (R_p_) and inhibition efficiency increase confirming their inhibitive action. The decrease in i_corr_ values can be due to the adsorption of the extracted molecules on the steel surface. *CgL* extract inhibits steel corrosion in 20% phosphoric acid solution more effectively than *Fen* extract at low extract concentrations of 0.4 g/L. Increased *Fen* extract concentrations above 0.4 g/L increase inhibition efficiency. The maximum inhibition efficiency for both extracts is about 80%. The significant change of βa and βc with *Fen* extract concentration indicates that *Fen* extract affects both anodic metal dissolution and hydrogen evolution reactions. The value of βc of *CgL* extract is around 120 mV, indicating that the proton discharge is the rate-determining step of the hydrogen evolution reaction.

### EIS measurements

**Figure 4:**
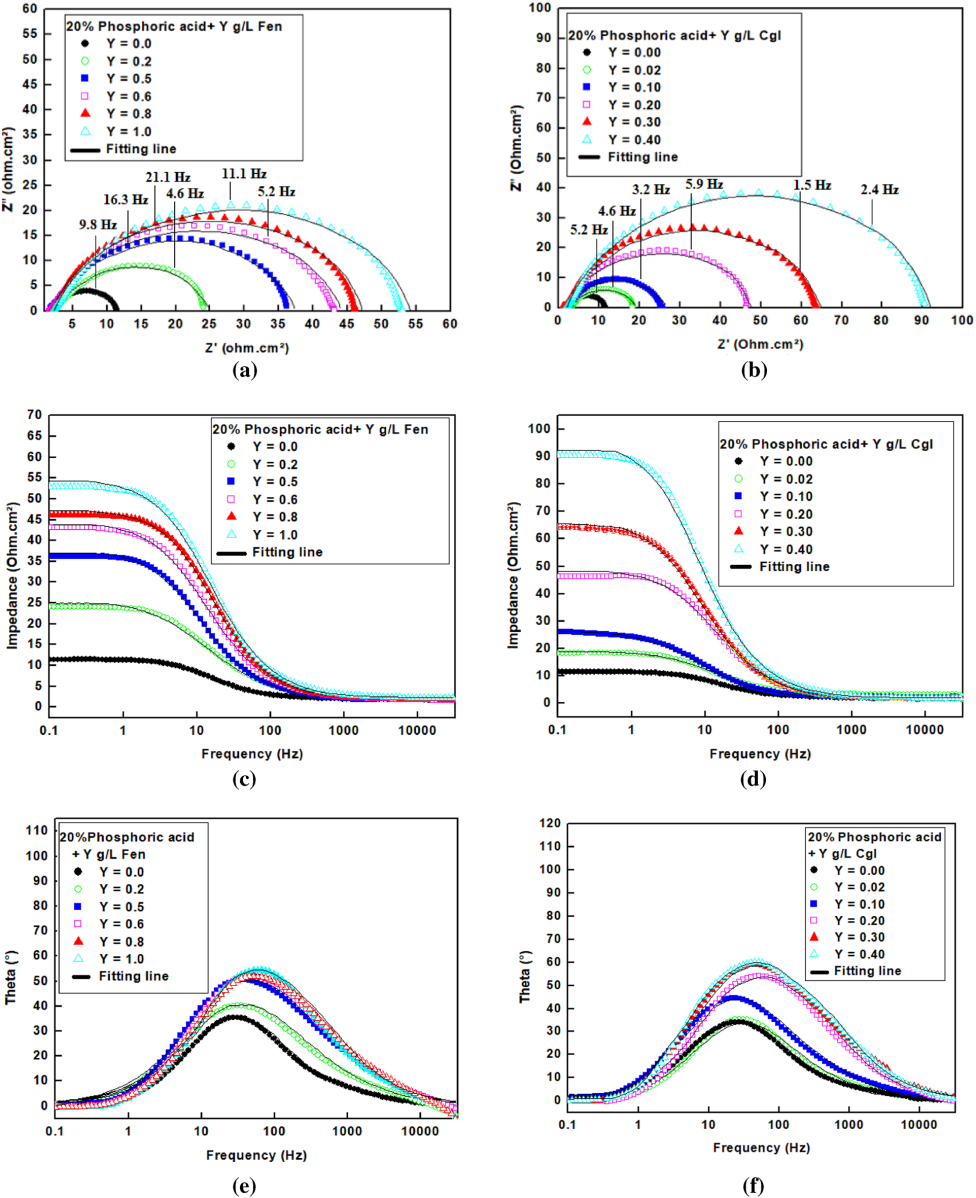
Nyquist, Bode and Theta plot of steel in 20% phosphoric acid in the absence and presence of *Fen* and *CgL* extracts.

Figure 4 depicts the Nyquist and Bode diagrams for *Fen* and *CgL* extracts. The Nyquist plots, Fig. 4a,b, are characterized by capacitive depressed semicircles signifying that steel corrosion is due to the charge transfer process^7^. The depressing characteristics may be attributed to the inhomogeneities and the surface roughness of the steel^30–33^. As the concentration of extracts increases, the diameter of the semicircles increases, so the corrosion rate decreases.

The Bode plots for steel in 20% phosphoric acid are depicted in Fig. 4c,d. The Figure shows that increasing the extract concentration leads to increasing the impedance magnitudes at the low frequency, confirming that the extract constituents form an adsorption film at the steel surface, decreasing the corrosion rate of steel^8^. The phase angle plots, Fig. 4e,f, show a one-time constant, which is attributed to the electrical double layer^34–36^. With increasing the concentrations of the extracts, the phase angle at the middle frequency regions was shifted to a higher value. This may be attributed to forming of a protective layer of the extract constituents at the steel surface, so the electrode interfacial structure was changed^2^.

**Figure 5:**
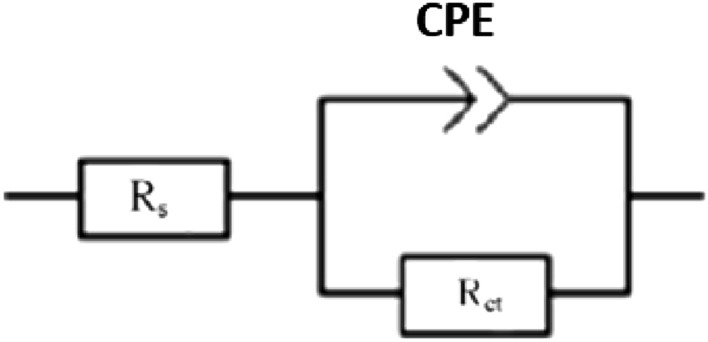
Equivalent circuit model.

The impedance parameters were accomplished by fitting the experimental data to the equivalent circuit shown in Fig. 5 using the Zsimpwin program. Table 3 displays the obtained impedance parameters.

**Table 3:** Electrochemical impedance parameters of steel in 20% phosphoric acid in the absence and presence of *Fen* and *CgL* extracts.

	Conc., g L^−1^	R_s_, Ω cm^2^	R_ct_, Ω cm^2^	Q_dl_, µF cm^−2^	n	C_dl_, µF cm^−2^	η	SD
*Fen* extract	0.00	1.86	10.64	1061	0.93	2142	–	–
	0.20	2.99	23.00	1068	0.92	2572	53.74	0.88
	0.40	2.52	28.68	1323	0.87	6394	62.90	0.56
	0.50	3.05	33.51	776	0.93	1668	68.25	0.48
	0.60	3.41	39.38	554	0.94	1048	72.98	0.32
	0.70	2.68	43.52	445	0.92	1049	75.55	0.41
	0.80	3.33	47.59	476	0.92	1138	77.64	0.24
	0.90	2.77	48.32	532	0.91	1452	77.98	0.22
	1.00	3.99	48.82	415	0.92	983	78.21	0.31
	1.20	3.88	50.00	458	0.92	1096	78.72	0.11
*CgL* extract	0.00	1.86	10.64	1061	0.93	2142	–	–
	0.02	3.41	15.51	1684	0.89	5919	31.40	0.41
	0.05	2.54	17.88	2077	0.92	5186	40.49	0.39
	0.10	2.50	22.95	1339	0.94	2589	53.64	0.35
	0.15	3.93	33.48	817	0.92	1986	68.22	0.28
	0.20	2.97	44.43	547	0.91	1485	76.05	0.21
	0.25	1.97	51.27	477	0.93	1020	79.25	0.18
	0.30	2.93	60.48	479	0.84	3390	82.41	0.11
	0.35	2.85	72.86	572	0.88	2440	85.40	0.08
	0.40	3.83	87.84	384	0.92	951	87.89	0.09

The solution resistances, Rs, represent the combined resistance of electrolyte and electrode material, which are nearly constant in the absence and presence of the extracts. The difference between the values of charge transfer resistance, R_ct_, and R_p_ determined by impedance and polarization measurements explains a complex reaction. The charge transfer resistance in a complex reaction is the point at which the mid-frequency loop intersects the real axis. R_p_ is the point on the real axis where the low-frequency loop intersects. The inhibition efficiency, η, is calculated from the charge transfer resistance, R_ct_, using Eq. (8)^37^.

8 $$\upeta = \left( {\left( {{\text{R}}_{{{\text{cti}}}} - {\text{ R}}_{{{\text{cto}}}} } \right){\text{/R}}_{{{\text{cti}}}} } \right) \times {1}00$$

where, R_cto_ and R_cti_ are the charge transfer resistances in the absence and presence of the extract, respectively. The double layer capacitance, C_dl_, could be calculated from the constant phase element (CPE) value of Q_dl_ and n using Eq. (9)^38^.

9 $${\text{C}}_{{{\text{dl}}}} = \, \left( {{\text{Q}}_{{{\text{dl}}}} \times {\text{ R}}_{{{\text{ct}}}} } \right)^{{{1}/{\text{n}}}} /{\text{R}}_{{{\text{ct}}}}$$

where n represents the deviation parameter. The n value varies between − 1 to 1; if n = − 1, it is revealed that the CPE is an inductor, while if n = 0, it implies that CPE is a pure resistor, as n = 0.5 this denotes the CPE is a Warburg impedance, and if n = 1 signifies the CPE is a pure capacitor^8^. The data in Table 3 shows that the value of n is lower than 1 by a small value indicating a small deviation of the CPE from being a pure capacitor. Increasing the R_ct_ in the presence of the extracts demonstrates the inhibitive action of the extracts takes place by the adsorption mechanism. The *CgL* and *Fen* extracts have maximum inhibition efficiencies of 87.89 and 78.72 at 0.4 g/L and 1.2 g/L, respectively. The decrease in C_dl_ indicates that the double layer thickness is increased, which leads to a decrease in dielectric constant because the extract's constituents displace the adsorbed water molecules at the steel surface^4,39,40^. The results of impedance measurements are nearly similar to those of polarization measurements.

### Potential of zero charge

**Figure 6:**
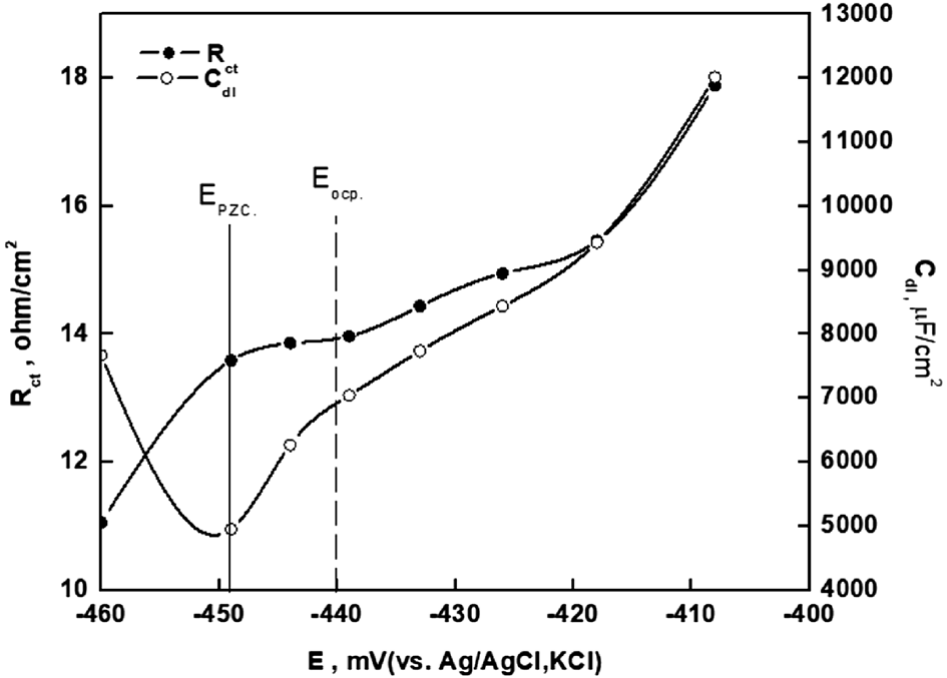
Variation of R_ct_ and Q_dl_ with the applied potential around the open circuit potential (− 440 mV) for steel in 20% phosphoric acid solution.

In electrostatic processes, the potential of zero charges (PZC) is an essential parameter. Figure 6 shows the variation of the double-layer capacitances calculated from the equivalent circuit previously described in Fig. 5 as a function of applied voltage. The PZC of a material in solution is the point at which minimum capacitance is obtained. The open circuit potential with respect to the PZC determines the metal's surface charge^27^. The open circuit potential (E_ocp_) and E_PZC_ values for steel in 20% phosphoric acid solution are − 440 mV and − 449 mV, respectively. Steel's surface charge (Er) is evaluated utilizing the equation Er = Eocp-Epzc, where Er is Antropov's rational corrosion potential^41^. The results show that the steel surface is positively charged (+ 9 mV) in a 20% phosphoric acid solution.

### Adsorption considerations

**Figure 7:**
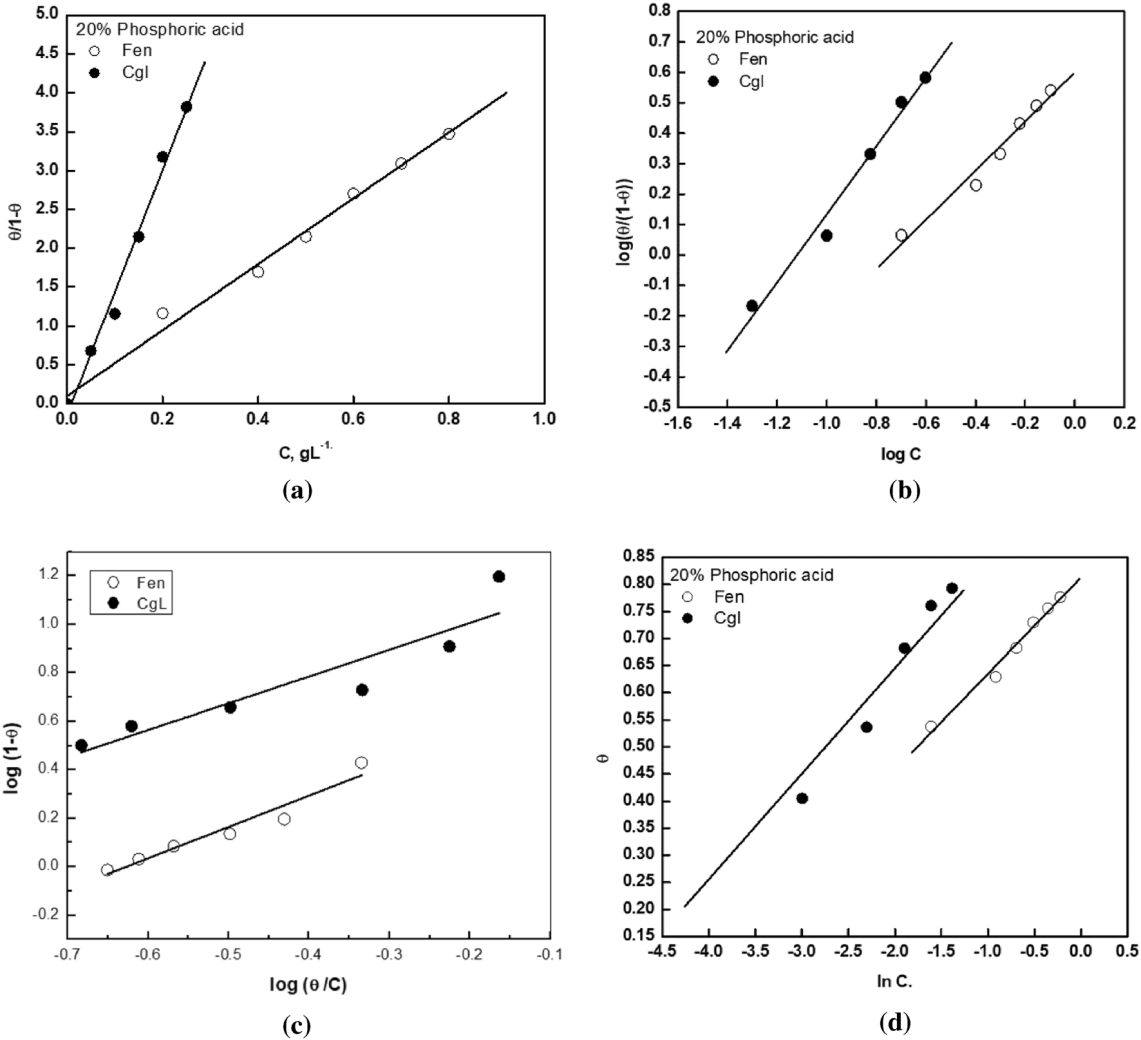
Application of (**a**) Langmuir, (**b**) kinetic-thermodynamic model, (**c**) Flory–Huggins, and (**d**) Temkin adsorption isotherm to the Fen and CgL extract adsorption results on mild steel surface in 20% H_3_PO_4_ at 30 °C.

Adsorption isotherms were applied to demonstrate the interaction between Fen and CgL extracts and mild steel surface in 20% H_3_PO_4_. Theoretical fitting of the Kinetic-thermodynamic model^42^ Langmuir isotherm, Flory–Huggins, and Temkin models^43^ are depicted in Fig. 7. The degree of surface coverage (θ = *η* × 100) values of the extract in 20% H_3_PO_4_ were obtained from EIS measurements.

Table 4 depicts the fitting parameters derived by fitting the experimental data to the examined isotherms. The ideal behavior of adsorption Fen and CgL extract at the steel surface is explained by the fitting of the experimental data to Langmuir adsorption isotherm and confirmed by the values of 1/y that indicates that a single inhibitor molecule occupies one active site. The ideal behavior of adsorption Fen and CgL extract at the steel surface was also confirmed by the size parameter (x) values that explain the number of water molecules displaced by one inhibitor molecule; its value equals the values of 1/y. These results confirm that the size of inhibitor molecules equals that of a water molecule. The Temkin isotherm shows that the positive value of the adsorption parameter (f) suggests mutual repulsion of molecules^44^. Moreover, as reported, the efficiency of a corrosion inhibitor is proportional to the magnitude of its binding constant K, i.e., large values of K mean better and strong metal-inhibitor interactions^45^. Thus, according to the numerical values obtained from Langmuir, kinetic–thermodynamic model, and Temkin isotherms, the binding constant K value for CgL extract is greater than that of Fen extract in H_3_PO_4_ solutions, which confirms the experimental results.

**Table 4:** Linear fitting parameters for the adsorption of Fen and CgL extracts on steel surface in 20% H_3_PO_4_ at 30 °C.

Extracts	Langmuir		Kinetic model			Flory–Huggins			Temkin		
	K	R^2^	K	1/y	R^2^	K	x	R^2^	K	f	R^2^
Fen	4.24	0.99	5.53	1.24	0.985	6.45	1.29	0.93	96.45	5.62	0.99
CgL	15.30	0.99	15.74	1.14	0.974	16.78	1.10	0.82	202.26	5.13	0.97

### Effect of temperature

Many studies have focused on the influence of temperature on steel corrosion in the phosphoric acid solution^46^. In the present work, we investigate the corrosion of steel in 20% phosphoric acid in the presence of 0.8 g/L *Fen* and 0.4 g/L *CgL* extract at 30 °C, 40 °C, 50 °C, and 60 °C.

**Figure 8:**
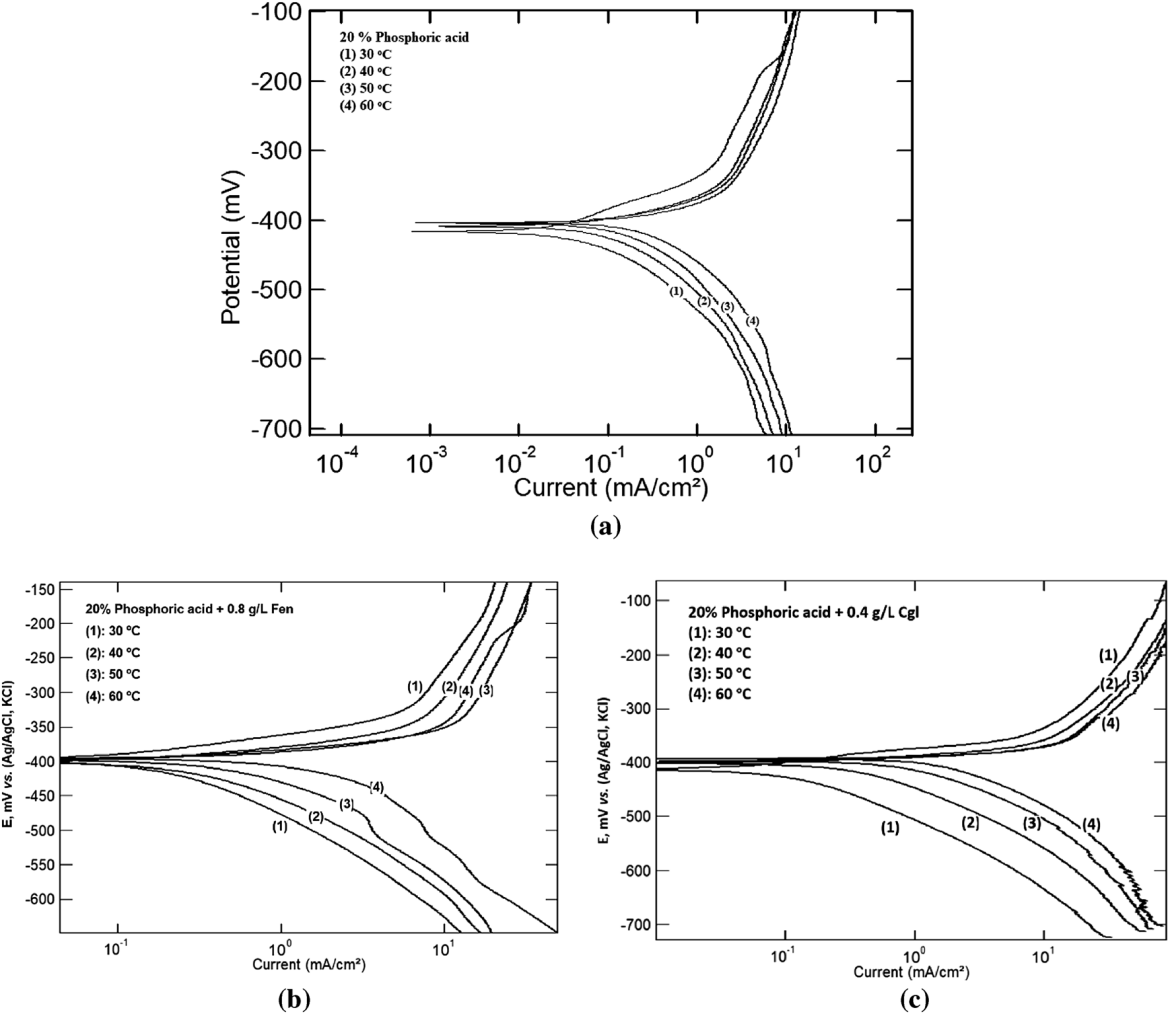
Potentiodynamic polarization curves of steel in 20% phosphoric acid in (**a**) the absence and presence of (**b**) 0.8 g/L *Fen* and (**c**) 0.4 g/L *CgL* extract at different temperatures.

The polarization curves in Fig. 8 show that increasing temperature enhances the hydrogen evolution reaction in the presence of both extracts. Furthermore, the temperature influences metal dissolution in the presence of *Fen* extract. The obtained electrochemical polarization parameters at different temperatures are shown in Table 5.

**Table 5:** Electrochemical polarization parameters of steel in 20% phosphoric acid in the absence and presence of 0.8 g/L *Fen* and 0.4 g/L *CgL* extract at different temperatures.

	Temp, °C	E_corr_, mV	βa, mV/decade	− β_c_, mV/decade	i_corr_, mA/cm^2^
Blank	30	− 493	307	149	6.20
	40	− 456	266	244	7.41
	50	− 459	283	212	8.88
	60	− 435	243	205	10.68
Fen extract	30	− 499	278	138	1.43
	40	− 483	281	173	2.32
	50	− 500	337	205	4.35
	60	− 472	450	211	5.72
CgL extract	30	− 517	189.95	110	1.2403
	40	− 487	186.27	110	2.4813
	50	− 471	224.17	111	5.0033
	60	− 465	238.93	120	7.1454

**Figure 9:**
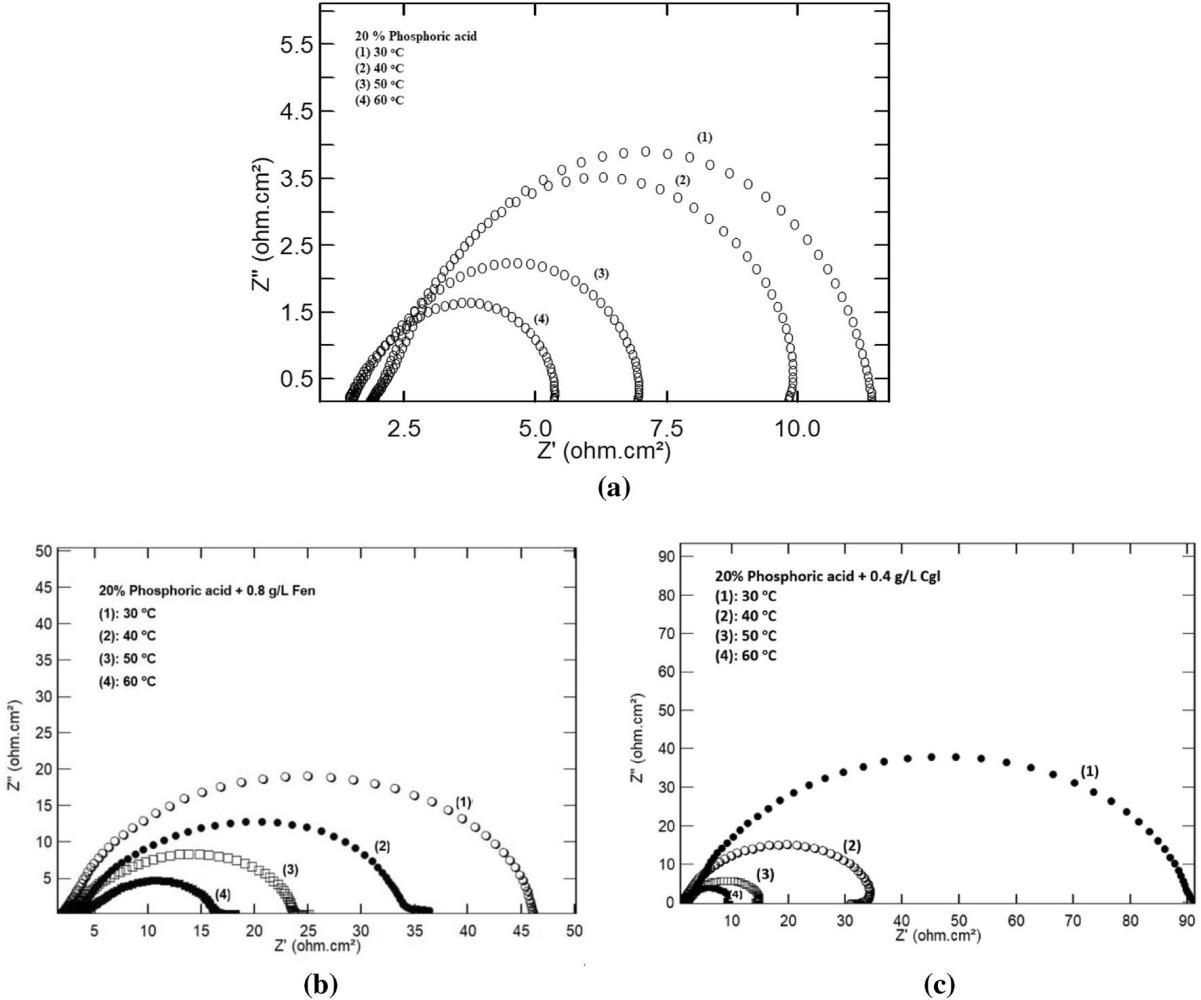
Nyquist impedance plot of steel in 20% phosphoric acid in (**a**) the absence and presence of (**b**) 0.8 g/L *Fen* and (**c**) 0.4 g/L *CgL* extract at different temperatures.

Figure 9 shows Nyquist diagrams of steel in 20% phosphoric acid in the presence of 0.8 g/L *Fen* and 0.4 g/L *CgL* extract at 30 °C, 40 °C, 50 °C, and 60 °C. As seen in the Figure, the Nyquist diagrams show a capacitive depressed semicircle signifying that the corrosion of steel is due to the charge transfer process; the size of the depressed capacitive semicircle diminishes as the temperature rises indicating an increased corrosion rate. The obtained electrochemical impedance parameters at different temperatures are depicted in Table 6. The values of R_ct_ decrease, and those of C_dl_ increase with increasing temperature. These are consistent with the physical adsorption of *Fen* and *CgL* extract molecules to the metal surface, with the adsorption–desorption equilibrium shifted toward desorption with increasing temperature.

**Table 6:** The EIS parameters of steel in 20% phosphoric acid in the absence and presence of 0.8 g/L *Fen* and 0.4 g/L *CgL* extract at different temperatures.

	Temp, °C	R_s_, Ω cm^2^	R_ct_, Ω cm^2^	Q_dl_, µF cm^−2^	n	C_dl_, µF cm^−2^
Blank	30	1.86	10.64	1061	0.93	2142
	40	2.11	8.05	1975	0.90	5786
	50	1.71	5.48	2973	0.89	9858
	60	1.57	4.01	3153	0.89	10,132
*Fen* extract	30	3.33	47.59	476	0.92	1138
	40	4.31	29.80	1295	0.90	4187
	50	3.66	20.27	1087	0.87	4844
	60	4.93	11.58	2836	0.90	9006
*CgL* extract	30	3.682	87.04	378.5	0.93	828
	40	2.34	32.84	559.4	0.95	938
	50	2.51	12.86	1782	0.96	2708
	60	1.98	8.11	2575	0.92	6115

The reciprocal of the absolute temperature 1/T has a linear relationship with the logarithm of the corrosion rate (k), according to many authors (Arrhenius equation)^47^:

10 $${\text{ln k}} = - {\text{ E}}_{{\text{a}}} /{\text{RT }} + {\text{ A}}$$

where E_a_ is the apparent effective activation energy, T is the absolute temperature, R is the universal gas constant, and A is Arrhenius pre-exponential factor.

**Figure 10:**
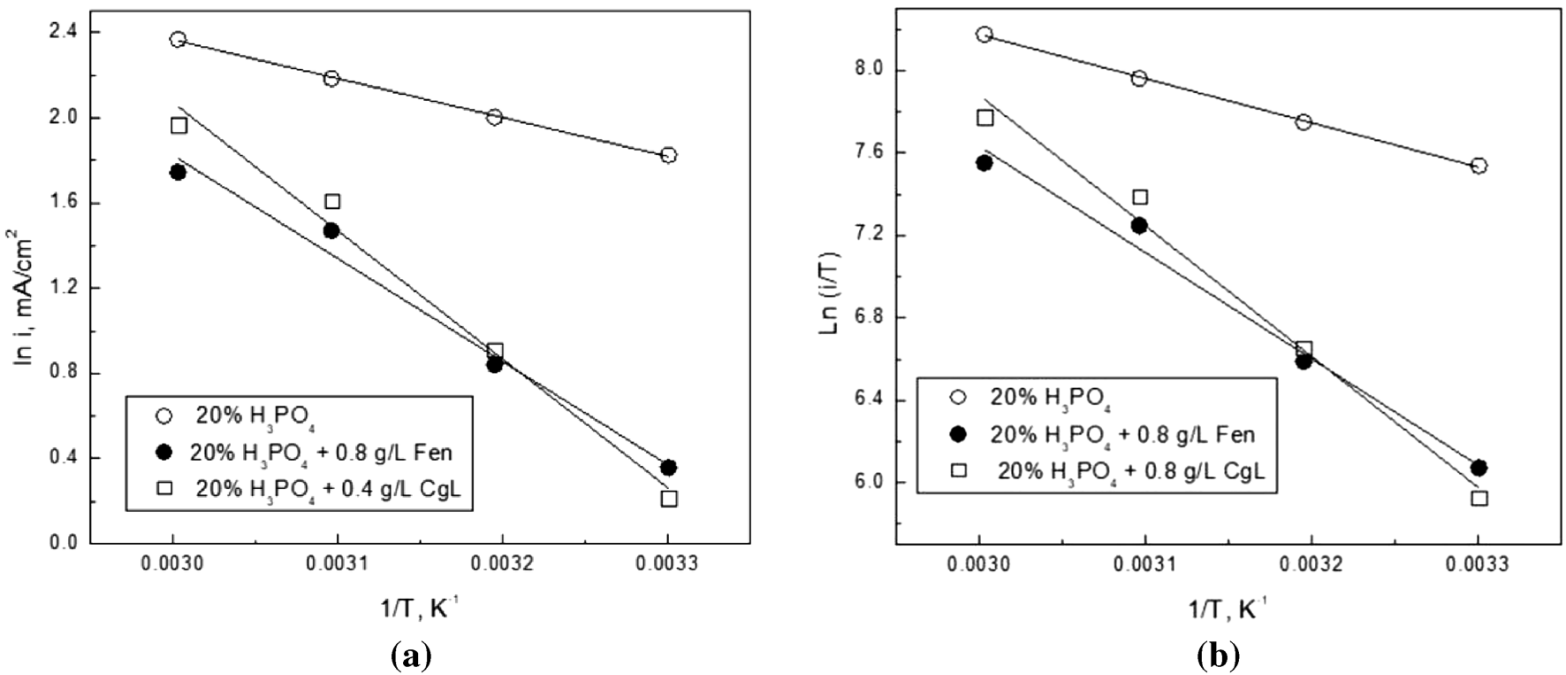
Variation of (**a**) lni and (**b**) ln i/ T vs 1/T for steel in 20% phosphoric acid in the absence and presence of 0.8 g/L Fen and 0.4 g/L CgL extract.

The transition state equation is an alternate formulation of the Arrhenius equation:

11 $${\text{k}} = \left( {{\text{RT}}/{\text{Nh}}} \right){\text{ exp }}\left( {\Delta {\text{S}}*/{\text{R}}} \right){\text{ exp }}\left( { - \, \Delta {\text{H}}*/{\text{RT}}} \right)$$

where N is Avogadro's number, h is Plank's constant, ∆H* is the activation enthalpy, and ∆S* is the activation entropy. The corrosion rates were taken as the corrosion current density. Figure 10 shows the plots used to determine the activation parameter of steel in 20% phosphoric acid in the absence and presence of 0.8 g/L Fen and 0.4 g/L CgL extract.

Table 7 displays the activation parameter values that were obtained. The variation in the activation energy values may be attributed to the alteration of the mechanism of the corrosion process in the presence of extract constituents^48^. The higher values of E_a_ and ∆H* in the presence of Fen extract indicate the extract's effectiveness at higher temperatures. The active complex denotes an association rather than a dissociation step, as shown by the negative value of ∆S*. As you progress from reactants to active complexes, you'll see a decrease in disordering. The entropy of activation changes signs from negative to positive due to the addition of the efficient *CgL* extract. Because the discharge of hydrogen ions to create adsorbed hydrogen atoms on the steel surface is the rate-determining step, the presence of efficient molecules will almost completely cover the surface, preventing the discharge process^49^.

**Table 7:** The activation parameters, obtained from polarization measurements, of steel in 20% phosphoric acid solution in the absence and presence of Fen or CgL.

	E_a_ (kJ/mole)	∆H*(kJ/mole)	∆S* (J/mole K)
Blank	15.19	17.83	− 76.16
Fen	40.24	42.88	− 5.40
CgL	50.10	52.74	+ 26.19

### SEM examination

**Figure 11:**
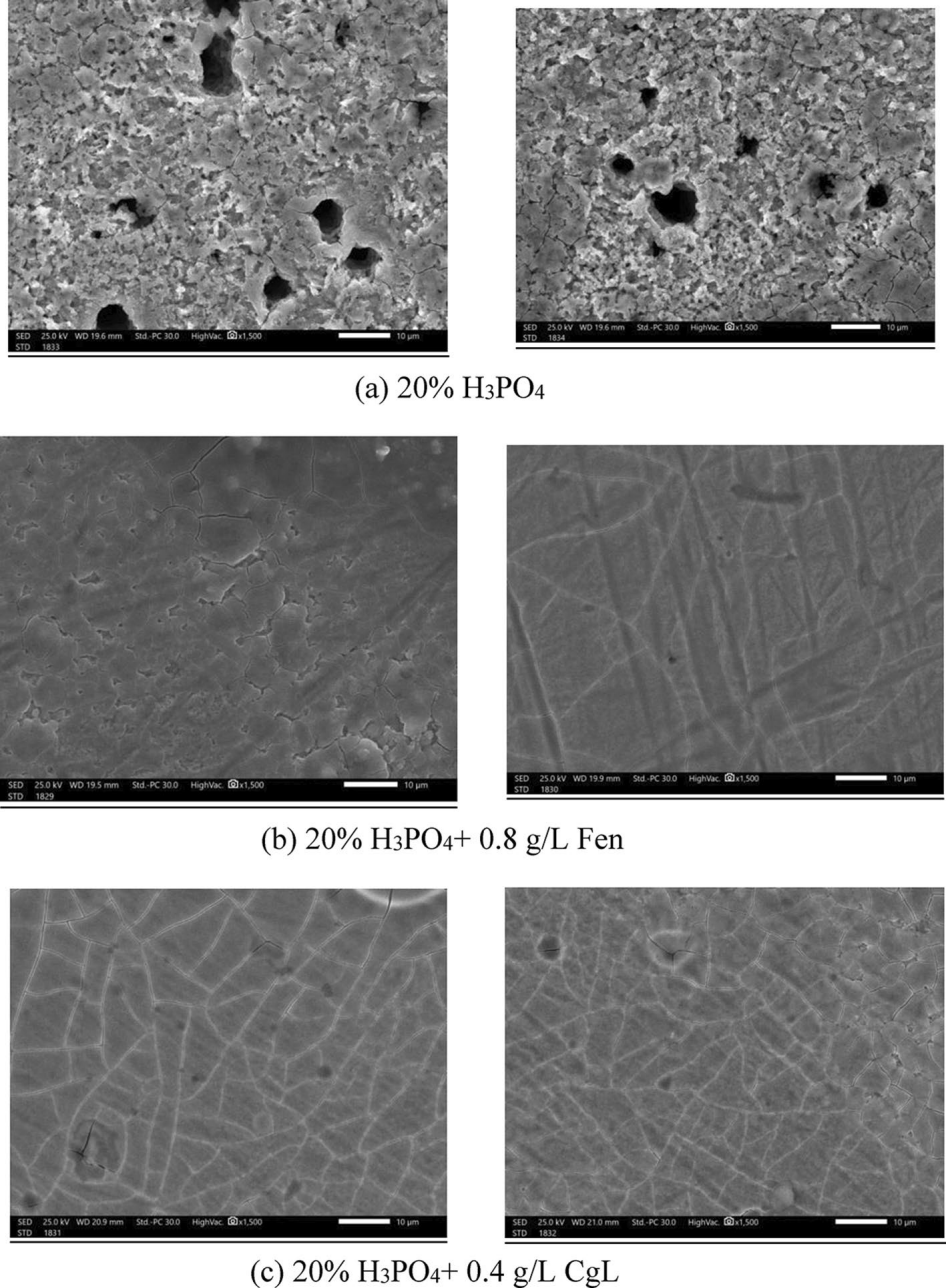
SEM micrograph of steel in the presence and absence of 0.8 g/L Fen and 0.4 g/L CgL extracts.

Figure 11 shows a SEM micrograph of steel in the presence and absence of 0.8 g/L Fen and 0.4 g/L CgL extracts. In the absence of extracts, Fig. 11a shows a rough surface with general corrosion and a big shallow pit, suggesting the phosphoric acid's vigorous attack on the steel surface. However, in the presence of Fen extract, Fig. 11b, the surface morphology becomes smooth, which may be related to the adsorption of chemical constituents of *Fen* extracts, blocking the surface roughness and shallow pits. Figure 11c also clarifies the formation of the smooth film due to the adsorption of chemical constituents of *CgL* extracts (Ferulic acid) over the steel surface. This behavior corroborated the results of polarization and impedance tests.

### Quantum calculation

**Figure 12:**
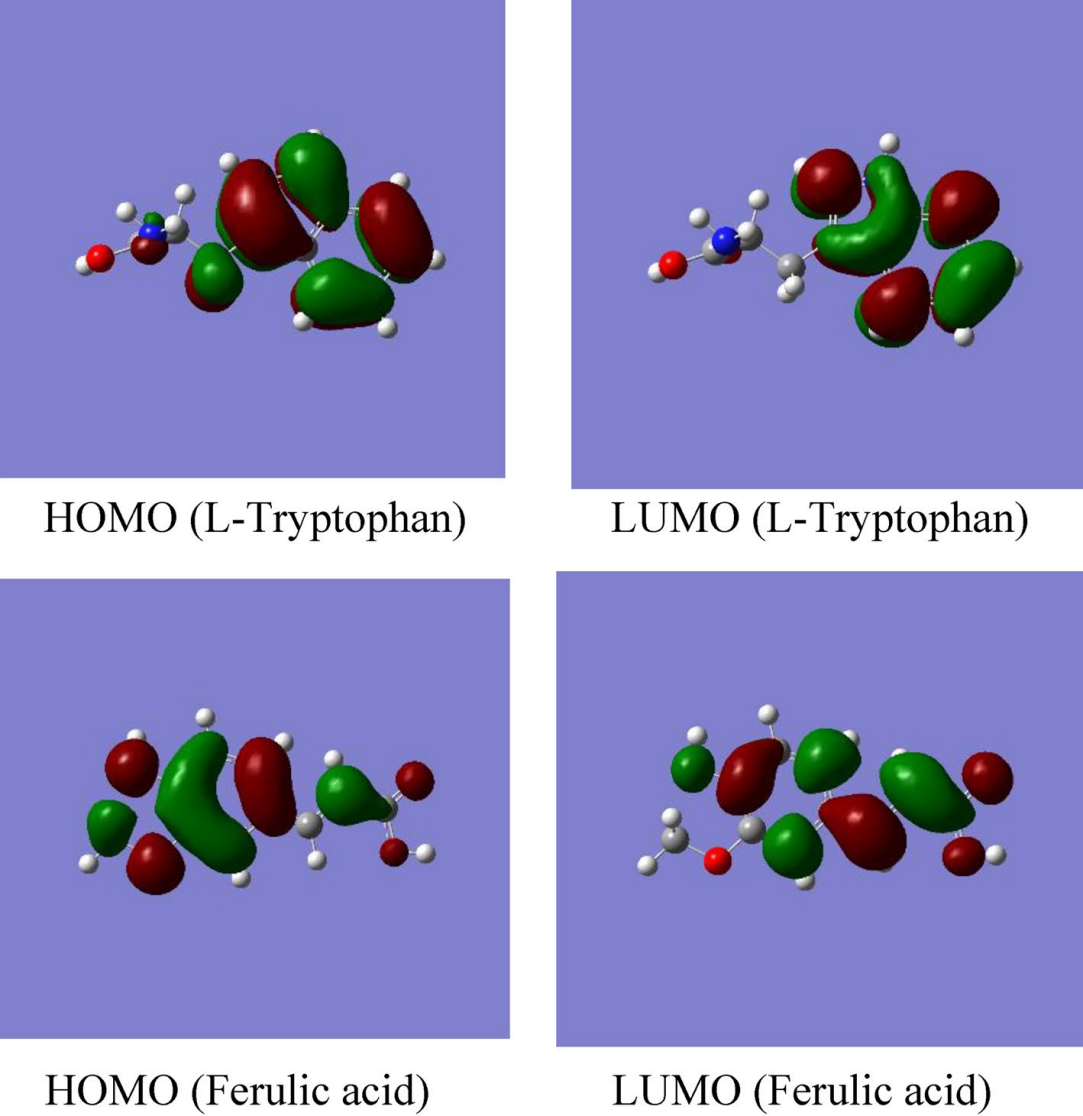
Optimized structure, E_HOMO,_ and E_LUMO_ of inhibitors for l-Tryptophan and Ferulic acid.

The use of quantum calculations in corrosion inhibitor research has received a lot of interest in the recent decade. Because of its theoretical background, quantum calculation-based density function theory (DFT) is widely accepted as a "green corrosion inhibition technology". The higher occupied molecular orbital and lower unoccupied molecular orbital energy, E_HOMO_ and E_LUMO_, of Ferulic acid and l-Tryptophan which are the major chemical constituents in CgL and Fen extracts, respectively, are shown in Fig. 12. Table 8 depicts the calculated quantum chemical descriptors for the optimized structure of Ferulic acid, and l-Tryptophan obtained using DFT with the B3LYP/6-311G(+) basis set in the aqueous phase^50^.

**Table 8:** Quantum chemical parameters (eV) for the optimized structure of Ferulic acid and l-Tryptophan obtained using DFT with the B3LYP/6-311G(+).

	E_HOMO_	E_LUMO_	Δ*E*	*I*	X	η	S
Ferulic acid	− 8.03	− 5.94	− 2.08	8.03	6.99	1.04	0.96
l-Tryptophane	− 8.31	− 4.77	− 3.53	8.31	6.54	1.77	0.56

According to Table 8, ferulic acid has less negative HOMO energy and a smaller energy gap (ΔE = E_HOMO_ − E_LUMO_), implying a stronger adsorption bond^51,52^.

The ionization potential (*I*) can be calculated using DFT-Koopmans' theorem as the negative of the highest occupied molecular orbital energy (I = − E_HOMO_).

Decreased ionization potential greatly affects Ferulic acid's adsorption and inhibitory characteristics. *X* signifies the absolute electronegativity of the inhibitor molecule (*Χ* = − ½[E_HOMO_ + E_LUMO_]*),* and *η* denotes the absolute hardness of the inhibitor molecule (*η* = *½* Δ*E)*. The ability of atoms in molecules to attract electrons is measured using electronegativity. The greater its value, the easier it is to attract electrons, indicating a stronger inhibition effect.

The softness parameter, S, is the inverse of the hardness parameter that measures the softness of the inhibitor and, thus, its reactivity. The most effective metal corrosion inhibitors are those with the highest softness^51^. This is because a soft molecule (inhibitor) is more reactive toward a metal surface than a hard molecule (inhibitor)^53^. Thus, ferulic acid with higher χ and S but lower *I* and η is a better corrosion inhibitor than l-Tryptophan. These observations are consistent with the results of the experimental measurements for *CgL* and *Fen* extracts.

### Inhibition mechanism

**Figure 13:**
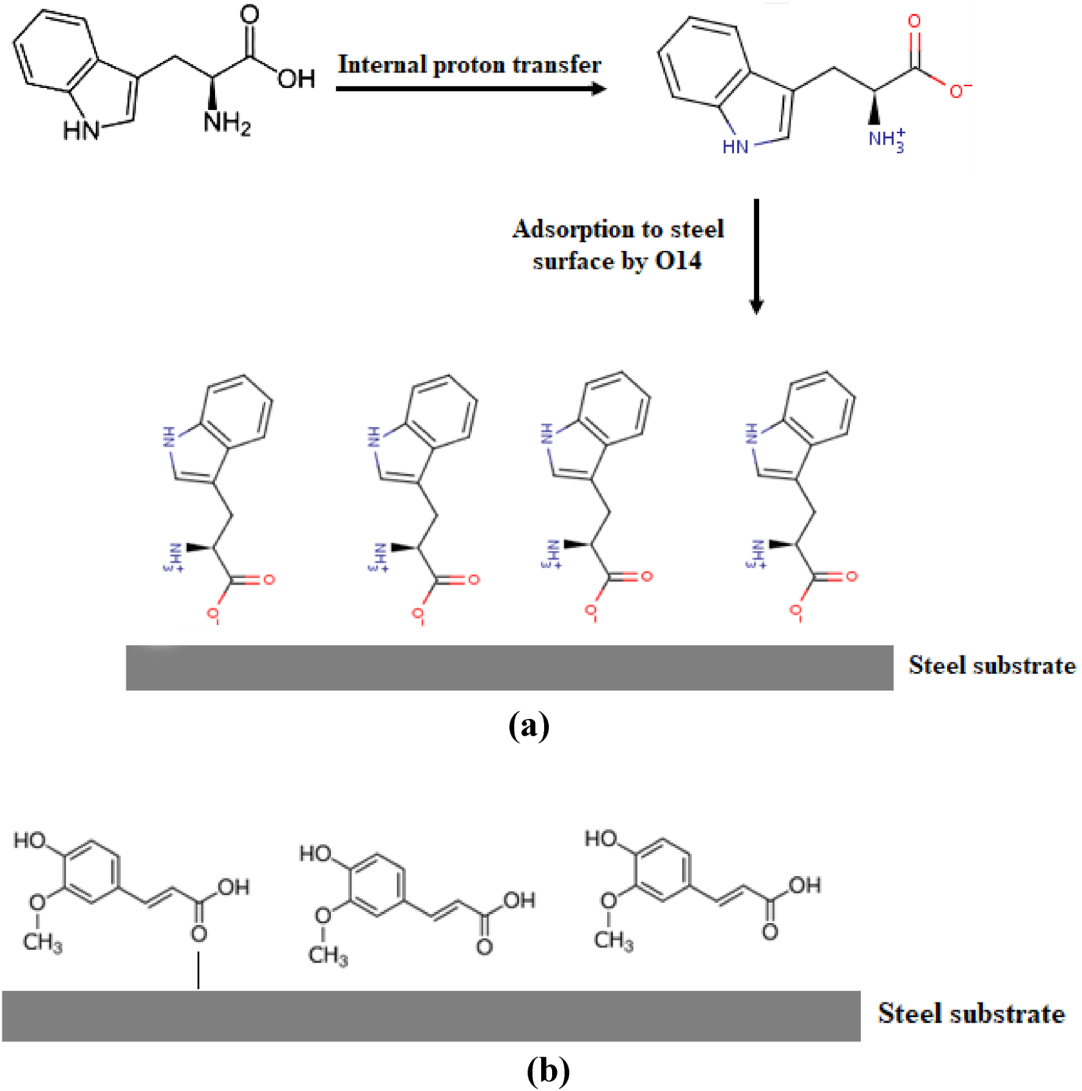
Schematic diagram of the corrosion inhibition mechanism of steel corrosion by (**a**) l-Tryptophan and (**b**) Ferulic acid.

To discuss the mechanism of inhibition of the major active ingredient of the extracts, it is crucial to calculate the Extended Huckel charge, Table 9, for the 3-dimensional structure of l-Tryptophane and Ferulic acid, which are the major chemical constituents in Fen and CgL extracts respectively, Fig. 13.

**Table 9:** Extended Huckel charges for nitrogen and oxygen atoms in l-Tryptophan and Ferulic acid.

l-Tryptophan		Ferulic acid	
N(2)	− 0.253	O(7)	− 0.203
N(7)	0.444	O(10)	− 0.225
O(14)	− 0.617	O(13)	− 0.692
O(15)	− 0.215	O(14)	− 0.179

l-Tryptophan zwitterion is generated by the internal transfer of a hydrogen ion from the –COOH group to the –NH_2_ group, resulting in an ion with both a negative and positive charge. The inhibition of steel corrosion by tryptophan zwitterion could be attributed to the adsorption of (O14) of the carboxylate group over the positively charged iron phosphate film. In comparison, the inhibition of steel corrosion by Ferulic acid could be attributed to the adsorption of (O13) of the carboxylate group over the positively charged iron phosphate film.

## Conclusions

1. Fenugreek seed extract (*Fen*) and Cape gooseberry leaf extract (*CgL*) act as eco-friendly corrosion inhibitors for mild steel in phosphate fertilizer manufacturing. The inhibition efficiency increases with the extract's concentrations reaching an inhibition efficiency of roughly 80% for 0.4 g/L *CgL* and 1.2 g/L *Fen* extracts.
2. The binding constant, obtained from the tested isotherms, indicated that *CgL* extract is a better and strong metal inhibitor than *Fen* extract. Increasing temperature increases the corrosion rate in the presence of both extracts suggesting a physisorption mechanism.
3. SEM micrograph of steel in the presence and absence of *Fen* and *CgL* extracts clarifies the formation of smooth film that can be due to the adsorption of chemical constituents of extracts.
4. The calculated quantum chemical descriptors for the optimized structure of Ferulic acid and l-Tryptophan indicated that ferulic acid is a better corrosion inhibitor than l-Tryptophan. These results confirm the potentiodynamic polarization and EIS results.

## Data Availability

The datasets used and/or analyzed during the current study are available from the corresponding author upon reasonable request.
